# View-invariant representations in ancestral cortex

**DOI:** 10.1126/sciadv.ady9659

**Published:** 2025-11-26

**Authors:** Milan Becker, Nimrod Leberstein, Mark Shein-Idelson

**Affiliations:** ^1^ School of Neurobiology, Biochemistry, and Biophysics, Tel Aviv University, Tel Aviv, Israel.; ^2^ Sagol School of Neuroscience, Tel Aviv University, Tel Aviv, Israel.

## Abstract

A multilayered, thalamorecipient visual cortex emerged ~320 million years ago in stem amniotes. Despite its importance for understanding the evolution of cortical computation, its function remains unknown. We recorded visually evoked responses in the dorsal cortex of behaving turtles, considered a mammalian neocortex homolog. Using a spatial oddball paradigm, we found tuning to stimuli in deviant positions alongside adaptation to standard positions within the visual field. Eye tracking demonstrated that responses remained spatially selective despite gaze shifts altering retinal stimulus position. Thus, the turtle cortex encodes unexpected visual stimuli using computations invariant to retinal position, a property previously observed only in higher mammalian cortices. These results indicate that invariance computations preceded the evolution of local filtering computations in mammalian primary cortices, pointing to a previously unidentified function for ancestral cortices. They also challenge hierarchical models of invariance computations, which assume that invariance is built from low-level features across multiple processing steps.

## Teaser

Turtle cortex encodes unexpected stimuli invariant to gaze, suggesting early evolution of cortical computation.

## INTRODUCTION

**Fig. 1.**
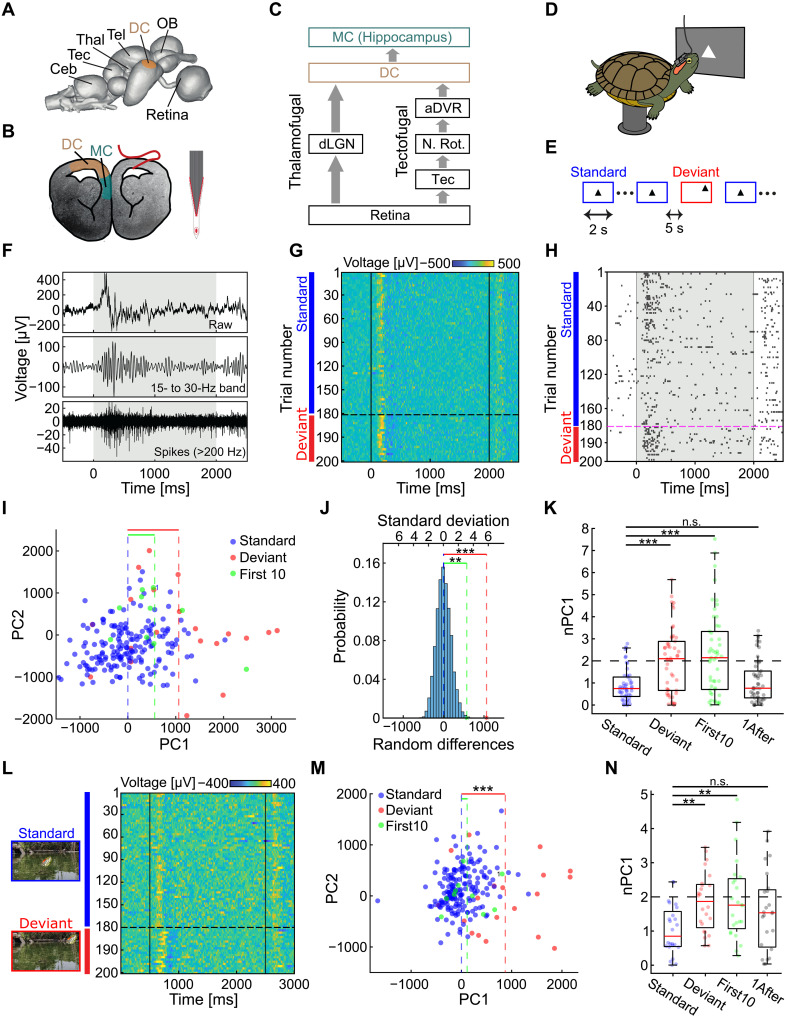
Visual cortex of behaving turtles exhibits spatial selectivity to novel stimuli. (**A**) Turtle brain surface. (**B**) Frontal cross section (magnetic resonance imaging) with implanted flexible probe (red illustration; probe design on the right). (**C**) Schematics of the two main visual pathways conveying retinal information to the pallium. Ceb, cerebellum; N. Rot., nucleus rotundus; OB, olfactory bulb; SC, superior colliculus; Tec, optic tectum; Tel, telencephalon; Thal, thalamus. (**D**) Schematic of the experimental setup. (**E**) Visual stimulation paradigm. (**F**) Electrode recording in response to visual stimulation (shaded areas). Top: raw data; middle: 15- to 30-Hz band; bottom: high-passed (>200 Hz) spiking activity. (**G**) LFP responses to consecutive trials during oddball stimulation. Stimuli are presented chronologically (every third standard trial) with deviant trials at the bottom. (**H**) Same as (G) but showing a raster plot (every second standard) of a putative single unit. (**I**) First two PCs for all LFP responses in one session [notice the separation between deviant (red), first 10 trials (green) and standard (blue) stimuli along PC1]. (**J**) Significance evaluation using a permutation test. Histogram of the mean-subtracted (all trials) mean PC1 over 20 trials compared with measured averages: standard (blue), deviant (red), and first 10 trials (green). (**K**) Summary statistics of the normalized (*z*-scored) PC1 for all recording sessions (*N* = 50 from 11 animals). Note differences between the standard (20 randomly selected trials) and the following trial groups: Deviant; First10; and 1After, the first stimuli after a deviant presentation. (**L** to **N**) Same as (G), (I), and (K) but for a naturalistic oddball experiment in which a turtle head emerges from a pond in different locations. An example for the stimuli is shown in [(L), left]. Responses are significantly stronger to the oddball location and the first 10 trials relative to standard location. *P < 0.05; **P < 0.005; ***P < 0.0005; n.s., not significant.

Stem amniotes, the first vertebrates to fully adapt to life on land, emerged ~320 million years ago, marking the beginning of the evolutionary trajectory leading to reptiles and mammals (*1*). Amniotes underwent substantial adaptations compatible with the new ecological opportunities on land and particularly the increase in visual range (*2*). These adaptations included the addition of ciliary eye muscles that dramatically increased accommodation accuracy and range (*3*, *4*); the appearance of the atlas-axis complex that allowed flexible head movements and gaze changes (*5*); the expansion of the dorsal telencephalon (*1*); the increase in sensory (specifically visual) inputs to the pallium (*6*, *7*); and the emergence and elaboration of new and existing areas including the claustrum and the layered dorsal cortex (DC). The latter is located in the dorsal telencephalon (Fig. 1A) and is considered to be a mammalian neocortex homolog (*8*–*11*). However, it remains unknown whether these adaptations were accompanied by new neural computations that may have shaped the evolution and function of the cortex.

Recent single-cell transcriptomic investigations considerably advanced our understanding of pallial homologies across amniotes (*9*, *11*–*13*). However, behavioral and neurophysiological investigations are required for placing the evolution of cell types within a computational context and unraveling the functional evolution of the cortex. Studies over more than 50 years attempted to do this by investigating the turtle DC (Fig. 1, A and B) (*6*, *7*, *14*–*16*). This three-layered cortex is a primary visual area that, as in mammals, receives direct inputs from both the thalamofugal pathway (via the dorsolateral geniculate nucleus, dLGN) and the tectofugal pathway (via the anterior dorsal ventricular ridge, aDVR) and directly projects to the neighboring medial cortex (MC), a hippocampal homolog (Fig. 1, B and C) (*6*, *7*, *9*). Behavioral and lesion experiments suggest that the DC is important for visual navigation (*17*), visual learning (*18*), and novelty detection (*19*). However, no physiological recordings from behaving animals exist to support these functions. Recent work in anesthetized turtles has provided valuable insights (*16*). This study showed that although DC receives direct input from the retinotopically organized dLGN, its neurons are not spatially nor orientationally tuned (*16*, *20*). Although DC neurons were found to lack defined receptive fields, they selectively adapted only to locations that were frequently stimulated, suggesting that local spatial information is encoded in DC activity (*16*). Furthermore, this spatially selective adaptation was not observed in the dLGN, suggesting that it originated in DC. These findings indicate that DC neurons exhibit tuning properties distinct from those of mammalian V1, but the functional role of the DC remains unknown.

One possibility is that the lack of localized receptive fields in DC is an artifact of the anesthetized state of previously recorded animals (*16*). Accordingly, retinotopic patterns, similar to those measured in the primary visual cortices of mammals (*21*, *22*), would be found in awake turtles. Alternatively, DC may engage in global computations across large parts of the visual field. Specifically, the spatially selective adaptation observed in anesthetized animals (*16*) raises the possibility that DC could be involved in the detection of newly appearing visual events requiring attention. For such computations to be effective, however, they must be view-invariant, that is, robust to the continuous changes in retinal input produced by gaze shifts, which alter activity in retinotopically organized brain areas. In primates, such invariance enables object recognition despite changes in viewpoint, size, orientation, or illumination (*23*, *24*). These computations are thought to emerge gradually along the ventral visual stream through pooling of low-level features across multiple retinotopic areas (*25*–*27*), consistent with the progressive enlargement of receptive fields along the visual hierarchy (*21*, *24*). Such view-invariant processing would likely benefit visual animals broadly and may be particularly important for turtles given their frequent head and eye movements (*28*). However, the existence of such computations in reptiles has not yet been investigated.

## RESULTS

### DC activity is tuned to visual novelty

In this study, we tested the hypothesis that the spatially selective adaptation in DC is view invariant. To do so, we first examined whether the spatially selective adaptation observed in anesthetized turtles (*16*) exists also in behaving animals. We performed recordings from DC (Fig. 1, A and B), using flexible 32-channel electrode arrays (Fig. 1B) stained with a lipophilic dye to verify recording locations post hoc (fig. S1) according to the turtle brain atlas (*29*). We placed turtles on a pedestal in front of a visual stimulation screen positioned contralaterally to the recorded hemisphere (Fig. 1D) and presented a spatial oddball paradigm, in which a triangle is repeatedly shown in one standard location (2-s duration, 5-s interval) and, on average, once in 10 trials in a different location (Fig. 1E). Consistent with previous studies (*16*, *30*), DC local field potential (LFP) was typified by a strong response after stimulus onset, followed by a longer oscillatory period. These transients were accompanied by an increase in spike rates (Fig. 1F). Visual inspection of LFP and spiking activity indicated that they were notably variable between trials but clearly different between standard and deviant trials (Fig. 1, G and H). To quantify these differences, we focused on the LFP signals, which were robust across experiments. We calculated the principal components (PCs) of the LFP waveforms during the visual response (Materials and Methods; fig. S2) and examined their values across trials. Deviant trials separated from standard trials along the first principal component (PC1; Fig. 1I), indicating that the difference between these groups constituted the direction of the largest response variance. The responses to the first standard trials (Fig. 1I, green) differed from the rest of the standard trials along the same PC as the deviant trials, suggesting that DC responses were driven by deviant stimuli.

To evaluate the robustness of our results and calculate their statistical significance, we used permutation tests (Materials and Methods; fig. S2). The average response intensities (PC1) for deviant trials (Fig. 1J, red dotted line) and for the first 10 trials in a session (Fig. 1J, green dotted line) were significantly larger (*P* < 0.0001 and *P* < 0.01, respectively, permutation test) than for randomly selected averages of 20 trials (Fig. 1J, blue bars). These results were consistent across recording sessions and animals. Analyzing single recordings separately revealed that the average PC1 of the deviants in most (28 of 50) sessions exceeded two standard deviations of the permuted distributions (Fig. 1K; *N* = 50 from 11 animals) with at least one recording session exceeding two standard deviations in most (10 of 11) animals (fig. S3). To analyze the significance over all recordings together (Fig. 1K; *N* = 50 from 11 animals), we normalized (*z*-scored) PC1 for each recording and compared their averages across stimuli groups. This revealed a significant difference between deviant and standard stimuli (*P* < 0.0001, permutation test) as well as between the first 10 trials and standard stimuli (*P* < 0.0001, permutation test).

To examine whether the responses were due to a general change in stimulus location rather than the novelty of the deviant location, we examined the response intensities of standard stimuli presented immediately after the deviant stimuli. These responses were not significantly higher than other standard stimuli (Fig. 1K; *P* > 0.3, permutation test). Furthermore, stronger deviant response intensities were observed for different combinations of standard and deviant positions on the screen (fig. S4) and were found across large spatial extents in DC (fig. S1). We next asked whether spatially selective adaptation was exclusive to the abstract shapes or whether it persists in a more visually complex setting. To test this, we used the same oddball paradigm, but with a conspecific turtle head emerging from a pond (the home pond of the test turtles) at different locations (Fig. 1L). In accordance with the results above, for this naturalistic setting (Fig. 1, L and M), both the deviant and first 10 trials showed significantly different responses from standard trials (Fig. 1N; permutation test: standard versus deviant, *P* < 0.002; standard versus first 10, *P* < 0.003; standard versus 1 after, *P* > 0.05; *N* = 24 from three animals).

To further exclude the possibility that differences in response intensity between the standard and deviant stimuli were due to biases in the response intensity across visual space, rather than adaptation to the standard stimulus, we presented stimuli appearing in random positions on a grid on the screen. While the positions were the same as those used during the oddball stimulus, all positions were presented randomly but with equal probability (fig. S5). For these spatial control sessions, rectangles (instead of the triangles) were used to minimize association with the oddball session. These experiments revealed that spatial biases could not explain the responses seen in the oddball experiment (fig. S5, D to F). This was also the case (fig. S5, G to I) when performing the same spatial control for the naturalistic stimulus (Fig. 1, L to N). For the naturalistic experiments, we used the same stimulus (turtle head) for both oddball and control experiments, indicating that the results of the spatial control are not dependent on using a different stimulus shape in oddball and control experiments. Last, we found that the stimulus offset response showed a considerably weaker but significant (*P* < 0.01, permutation test, *N* = 50 from 11 animals) difference between standard and deviant stimuli (fig. S6). Together, our results indicate that selectivity to the onset of stimuli in a deviant location, for both abstract and natural stimuli, is a dominant feature of visual responses in the DC of behaving turtles. Thus, in accordance with previous behavioral studies (*19*) and anesthetized neuronal recordings (*16*), DC responses in awake turtles are sensitive to novelty in the visual environment.

### Novelty responses are invariant to gaze shifts

**Fig. 2.**
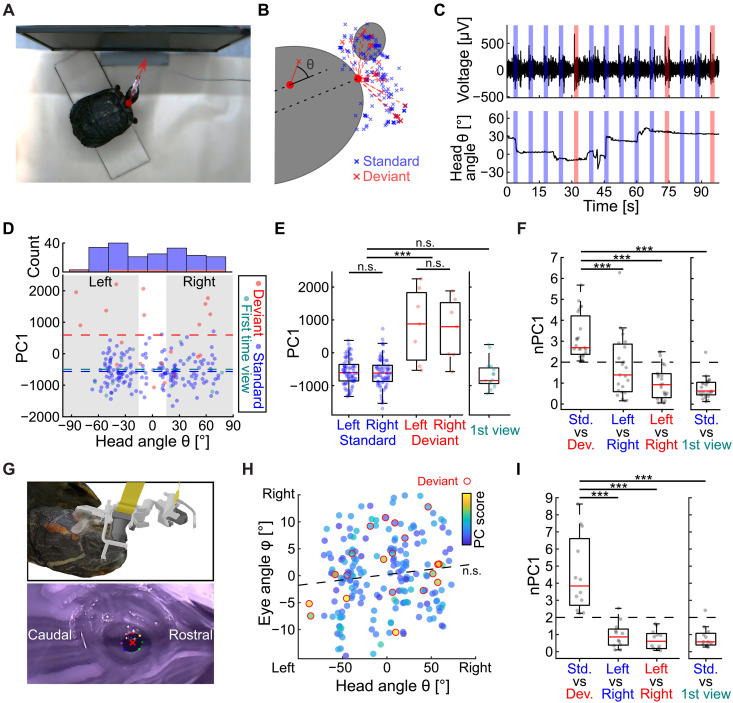
Visual spatial selectivity is invariant to gaze direction. (**A**) Top view of a turtle during an oddball experiment with annotated head direction (red arrow). (**B**) Head direction during standard (blue) and deviant (red) stimulations. Turtle carapace (cut ellipse) and head position during one trial (small ellipse) are shown in gray. (**C**) Raw electrode data (top) and head angles (bottom), during oddball visual stimulation (shaded area). Note increased response to deviant (red) relative to standard (blue) stimuli despite substantial changes in head angles. (**D**) Response intensity (PC1, as in Fig. 1I) as a function of head angle for standard, deviant, and first view trials in which an angle was viewed the first time (0° defined as average angle, Θ < −15° left direction, and Θ > 15° right direction). Dashed lines mark the average values. Top: A histogram across head angles. (**E**) Statistics over trials in (D). Note significant difference between standard and deviant trials, but not between left and right, or between standard and first view trials. (**F**) Statistics of the normalized (as in Fig. 1K) response intensity over sessions; the difference between standard (Std.) and deviant (Dev.) is significantly bigger compared to differences between left and right viewing angles and between standard and first-time viewing. (**G**) Three-dimensional (3D) model of the head-mounted gaze-recording system (top) and the eye images it acquires (bottom) with segmented pupil contour (colored dots) and center (red x). (**H**) The horizontal eye versus head angle across trials with color-coded response intensity (red outline marking deviant stimuli). Head angles are not compensated for by eye angles in the opposite direction (linear regression, dotted line), and both deviant and standard stimuli are found across head and eye angles. (**I**) Summary statistics over sessions as in (F) but with the estimated viewing angle (combining eye and head angles). (A) to (E) and (H) are from the same recording.

The consistent differences between the responses to the standard and deviant stimuli were surprising given that the turtles constantly moved their heads during recording sessions. In anesthetized animals (*16*), the decreased responses to the normal stimuli could be explained by long-term adaptation in the corresponding retinotopic position. In contrast, head movements are bound to change retinal illumination patterns for both the deviant and normal stimuli. To verify that retinal stimulation changed during trials, we tracked head movements (Fig. 2A and movie S1). Head position considerably varied between trials during presentations of both standard and deviant stimuli (Fig. 2B). By comparing neurophysiological responses for different head positions (Fig. 2C and movie S1), we observed that the deviant stimuli exhibited consistently higher response intensities relative to standard stimuli across all angles (Fig. 2, D and E). In contrast, when comparing response intensities during left (Θ < −15°) versus right (Θ > 15°) head angle groups, no such differences were observed, indicating that response differences between standard and deviant positions are invariant to head angle (Fig. 2, D and E; permutation test: standard versus deviant, *P* < 0.0001; left versus right, *P* > 0.7). Alternatively, DC could adapt to the standard stimulus separately for each viewing angle. In this scenario, we would expect a large response to a stimulus each time the turtle moves its head to a new position. However, examining the first responses to stimuli viewed from a new viewing angle revealed that this was not the case (Fig. 2D, green). Rather, first-view responses were not different from standard responses (Fig. 2E; *P* > 0.2, permutation test). The above results were consistent across recording sessions (Fig. 2F; *N* = 23 from eight animals) with differences between standard and deviant trials significantly higher compared to right versus left responses (*P* < 0.0005, permutation test) and to standard versus first-view responses (*P* < 0.0001, permutation test). Together, these experiments indicate that DC exhibits spatial selectivity that is independent of head direction.

Another possible explanation for the response invariance to head angles is compensation of head movements by eye movements such that the summed gaze angle remains constant throughout the experiment. In this scenario, standard and deviant stimuli could consistently excite the same retinal locations and adapt with known mechanisms (*31*). To test this, we developed a specialized head-mounted camera system (Fig. 2G, top; Materials and Methods) that allowed us to track the eye angle (Fig. 2G, bottom) in addition to the head angle. We found no compensatory correlation between eye and head angles at stimulus onsets (Fig. 2H; *P* > 0.08, Pearson correlation) (see fig. S7 for population analysis). Furthermore, both standard and deviant stimuli were observed across gaze angles (combining both head and eye) with deviant stimuli (Fig. 2H, red contour) consistently recruiting stronger response intensities. These results were echoed in the statistical analysis across sessions (*N* = 12 from three animals) with the left (Θ < −15°) and right (Θ > 15°) viewing-angle groups exhibiting no significant differences in responses, in contrast to the significantly higher differences between standard and deviant stimuli (Fig. 2I; *P* < 0.0001, permutation test). In addition, analysis based on both head and eye angles gave consistent results with the analysis based on head angles alone (fig. S8), further validating our head angle analysis (Fig. 2, C to F). Last, we verified that there was no behavioral difference during viewing standard versus deviant stimuli that could explain the difference in response intensity. Specifically, neither head nor eye movement speeds were different during standard versus deviant trials (fig. S9). Together, our data indicate that the differential DC response to standard and deviant positions is invariant to viewing direction.

### Large changes in retinal input due to self-motion recruit weaker responses than small but unexpected stimuli

**Fig. 3.**
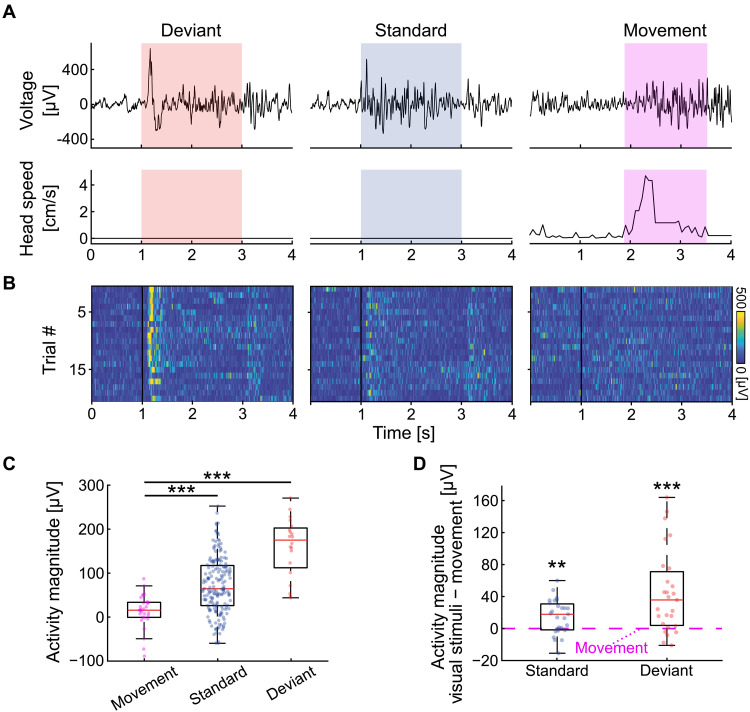
Head movements elicit weaker cortical responses than visual stimulation. (**A**) LFP traces (top) and head movements (bottom) recorded during deviant stimulus (red), standard stimulus (blue), and head movement (pink) from the same recording. (**B**) LFP heatmaps showing 20 responses to deviant stimuli (left), standard stimuli (middle, randomly selected), and movement (right, randomly selected) from the recording in (A). (**C**) Relative response magnitude, calculated as mean absolute LFP amplitude above baseline for all events in the recording in (B) (Materials and Methods). Notice significant differences between movement to deviant and to standard. (**D**) Relative response magnitude [as in (C)] for the response to standard and deviant stimuli relative to movement epochs (pink dashed line) over recording sessions. Notice that both the deviant and the standard stimuli elicit significantly stronger responses than movement across sessions (*N* = 27 from nine animals).

DC was previously shown to be strongly activated by natural images and videos (*16*). In such stimuli, retinal input dramatically changes at the time of image presentation. We therefore examined whether similarly strong responses would occur during head and eye movements (without visual stimuli), which considerably change retinal input across the visual field, especially during large movements (*28*). To assess this, we compared DC responses to head movements with the responses during oddball experiments (Fig. 1, D and E), in which light intensity changes in a relatively small area of the retina and should result in comparatively smaller retinal excitation (Fig. 3A). We did not find strong DC responses during episodes of movement without visual stimuli (Fig. 3B). Furthermore, presentation of deviant and standard stimuli recruited significantly stronger responses relative to the activity baseline (preceding the stimuli) than head movements (Fig. 3C; permutation test: deviant, *P* < 0.0001; standard, *P* < 0.0002). To verify these results over recordings, we subtracted the responses during movement from the responses during both standard and deviant stimuli for each recording session, resulting in significantly positive values (Fig. 3D; *t* test: standard versus movement, *P* < 0.002; deviant versus movement, *P* < 0.0002; *N* = 27 from nine animals). These results were consistent when comparing eye movements to visual responses (fig. S10). Thus, large changes to retinal input do not elicit strong DC responses when there is no novelty in the animal’s allocentric visual environment.

## DISCUSSION

Our findings shed light on the computational role of the DC, a reptilian homolog of the mammalian neocortex (*7*, *9*, *11*–*13*), offering fresh insights into the evolutionary origins of cortical processing. The lack of spatial tuning and retinotopic organization (*15*, *16*) complicated our understanding of the functional equivalence between DC and mammalian cortex. We bridge this gap by finding that DC encodes spatial novelty (Fig. 1) using a computation that is invariant to gaze direction (Fig. 2). Visual stimuli appearing in novel positions consistently evoked stronger responses than stimuli in expected positions, even though gaze shifts frequently altered the location of all stimuli on the retina. Intriguingly, the DC responded more strongly to these small but novel stimuli than to larger changes in visual input caused by self-motion. Together, these findings suggest that the ancestral cortex of stem amniotes was already capable of invariant sensory computations.

This idea is compatible with an ethological view of the evolution of the cortex. It is unlikely that the first ancestral visual pallial areas held representations similar to mammalian primary sensory cortex. Such retinotopically tuned neurons change their activity with every head and eye movement. From an ethological perspective, retinotopic representations carry little value without additional computations that can extract behaviorally relevant entities (e.g., the animal’s home or a predator) invariant to their projected position or size on the animal’s retina. Correspondingly, its activity reports the existence of an unexpected stimulus independent of gaze direction and therefore invariant to retinal excitation. This suggests that ancestral cortices may have initially evolved the capacity to compute invariance. If so, the local filtering properties observed in mammalian V1 did not exist in the ancestral cortex of stem amniotes but evolved later, possibly to refine computations of invariance. What then is the mammalian analog of DC?

Invariant computations are progressively abundant along the mammalian visual hierarchy (*26*), from retinotopic representations with little spatial invariance in V1 to highly invariant abstract representations in the medial temporal lobe (*32*). This hierarchical network is thought to combine simple local filters into increasingly complex objects (e.g., faces) that satisfy perceptual constancy to properties such as size, distance, rotation, and illumination. In the turtle, however, DC is both a primary visual area receiving input from the retinotopically organized dLGN (*16*, *20*) and, at the same time, the end of the cortical visual hierarchy (Fig. 1, B and C), forming a continuous three-layered structure with MC (the homolog of the hippocampal formation; Fig. 1B) (*6*, *9*, *12*). Thus, the invariant representation and the lack of cortical hierarchies in turtles challenge this hierarchical model and suggest that an alternative scheme for invariance computation (e.g., within a recurrent scheme) may exist.

View-invariant representations in turtle DC suggest functional analogy to medial temporal areas at the end of the mammalian visual hierarchy (Fig. 1C) (*33*). Specifically, hippocampus-adjacent regions such as the perirhinal and postrhinal cortices have been implicated in visuospatial learning. For example, in both rodents and primates, activity in these regions is tuned to specific objects (*34*) and is correlated with gaze changes (*35*). Since objects may change in appearance across viewing angles, reliable object identification requires invariance computations. In contrast, early visual areas in both mice (*36*) and monkeys (*37*) are highly sensitive to gaze shifts. Such sensitivity is absent from turtle DC during free viewing (without changes to the visual environment) (Fig. 3).

Another prominent DC feature observed in mammalian medial temporal areas is the increased responses to novel stimuli (*38*, *39*). In humans, similar responses to unexpected stimuli (mismatch negativity) were extensively studied (*38*) and are thought to subserve preferential memory encoding of salient events (*39*). This idea is consistent with strong DC oddball and first trial responses (Fig. 1) and weak self-movement responses (Fig. 3). Strong responses could be responsible for updating spatial information about the visual world and are not needed for expected visual input during self-motion. Mismatch negativity is not exclusive to mediotemporal areas but is also observed in primary sensory areas (*40*–*44*). These responses were suggested to reflect predictive coding computations that are shared across cortical areas (*45*–*47*). It is tempting to think that similar predictive computations exist in turtles: A network that predicts visual input given changes in viewing direction and updates when predictions deviate from sensory input may support the formation of an internal model of the visual environment (*48*, *24*, *49*). Such a mechanism could facilitate filtering nonsalient visual changes and robust identification of novelty in allocentric coordinates (*50*). It could also enable temporal prediction of future events, allow the circuit to adapt to dynamic regularities, and enable identification when they change (*47*, *51*).

Beyond sensory processing, DC may serve a role in navigation. MC, which forms a continuum with DC (Fig. 1, B and C) (*9*, *12*) and is densely interconnected with it (*6*, *9*), has been shown to play a role in map-based navigation (*52*, *53*). Moreover, the presence of place cells in the MC of both mammals (*54*) and avian reptiles (*55*) suggests that they may also have existed in stem amniotes. If so, the construction of an allocentric map in MC would greatly benefit from view-invariant processing, such as identifying landmarks despite movement. According to this hypothesis, spatial invariance transformations may have evolved to support cue-based navigation in the complex terrestrial visual environments encountered by stem amniotes (*1*, *2*). This processing scheme could have extended the egocentric saliency map in the optic tectum (*56*). However, this hypothesis remains speculative and awaits population recordings from the MC of behaving reptiles. Such measurements will also be critical for revealing how invariant computations are ultimately implemented in DC. The small size, relative simplicity, and unique ex vivo eye-brain preparations of the turtle cortex (*57*) highlight it as a valuable system for studying the neural basis of visual invariance and its evolution.

## MATERIALS AND METHODS

### Animals

Red-eared slider turtles (*Trachemys scripta elegans*) were kept in an enclosed outdoor pond at the zoological garden of the Tel Aviv University (approval number: H10900/2024). Before the experiments, animals (weight: 800 to 2000 g) were moved to an indoor tank [100 cm by 120 cm by 74 cm (width by length by height)] in the laboratory containing a basking platform and heat and light sources set to a 12-hour/12-hour light/dark cycle. All procedures complied with relevant ethical regulations for animal use. Ethical protocols were approved by the Tel Aviv University ethical committee (approval numbers: TAU-LS-IL-2410-145-4 and TAU-LS-IL-2411-147-4).

### Surgery

Twenty-four hours before surgery, analgesics (carprofen, 2 mg/kg) and antibiotics (enrofloxacin, 5 mg/kg) were administered. Animals were anesthetized using an initial intramuscular injection (ketamine, 0.3 ml/kg, and medetomidine, 0.4 ml/kg). After the animal stopped responding to touch (usually ~1 hour after injection), it was intubated and connected to a ventilation system (Anesthesia WorkStation 100, Hallowell EMC) maintaining a constant flow of 4% isoflurane to ensure continuous anesthesia (respiratory rate: 6 bpm, pressure: 6 cmH_2_O). The turtle was placed in a stereotaxic apparatus (RWD 68409). Body temperature was maintained at 30°C using a heating pad attached to the stereotaxic table. Eyes were protected by covering with ointment (Duratears). The skin covering the skull was disinfected with povidone-iodine 10% and coated with a lidocaine ointment (2%) for local analgesia. The skin on the skull was removed with a scalpel, and residual tissue was removed with a micro curette and dissolved with 30% hydrogen peroxide. A small craniotomy (~2 mm by 5 mm) was drilled using a dental drill. Four to five screws were screwed into regions surrounding the craniotomy to ensure anchoring of the dental cement to the animal. The dura and arachnoid meninges covering the forebrain were removed with fine forceps. Because the DC lies above a large ventricle, we used ultraflexible neural probes custom fabricated by NMI (Germany) to achieve stable long-term recordings in awake animals. Specifically, probes contained 32 channels organized in a V-shape (620 μm long) (Fig. 1B) made from porous, low-impedance (below 100 kilohm at 1 kHz), TiN electrodes (400 μm^2^ in area) embedded in ultrathin (6 μm) polyimide. After visually identifying the brain area of interest, the pia was gently opened with fine forceps, and the electrode array was inserted into the DC at an angle of around 45° and to a depth of ~700 μm such that all electrodes entered the cortex. An additional chlorinated silver wire (catalog #786000, A-M Systems) was placed in the cerebrospinal fluid for referencing. The craniotomy was closed using dental cement, which also fixed the electrode’s printed circuit board and omnetics connector to the skull. The printed circuit board was laid down horizontally to ensure that the animal could retract the head into its carapace, and a metal ring was embedded into the dental cement to enable pulling the animal’s head out of the shell to connect it to the headstage. In eye camera recordings, an additional M3 screw nut was anchored to the cement to enable mounting the eye-camera system. The animal was then placed under a heat lamp. Experiments started after the animals recovered from the surgery and exhibited normal behavioral patterns.

### Tissue staining and visualization

The back of the electrode arrays was coated with either DiI or CM-DiI (Invitrogen) for tracing electrode trajectories. The brain was extracted postmortem and fixated in 4% paraformaldehyde solution. Brains were inspected under a fluorescence macroscope (Axio Zoom V16, Zeiss) to identify the insertion site (clearly visible in all animals). In addition, in a subset of animals (*N* = 3), brains were cleared (iDISCO+ protocol version 0.9, https://idisco.info/) (*58*) to visualize insertion in 3D (fig. S1). The cleared brains were scanned in a 3i Cleared Tissue LightSheet microscope at two wavelengths: 488 nm for autofluorescence and 560 nm for visualizing the stained insertion site.

### Experimental setup

Animals were moved from their home tank into the recording tank for the duration of each experiment. This tank was the same size as the home tank but did not contain water and was enclosed by a Faraday cage. For the duration of the recording sessions, the carapace was fixed on a pedestal using Velcro tape. This allowed the turtles to freely move their limbs and head without moving their body (Fig. 1D). Turtles were placed ~15 cm away from a visual stimulation screen and rotated at ~30° such that stimulations fell on the eye contralateral to the implanted hemisphere (Fig. 2A).

### Electrophysiology

Animals were connected to a headstage (Intan RHD2132, #C3314) with an ultrathin serial peripheral interface (SPI) cable (RHD2000, #C3216), and neural signals were recorded (at 20 kHz) with an Open Ephys acquisition board (v2.2) and Open Ephys GUI software (*59*) and stored on a personal computer.

### Visual stimulation

All visual stimuli and paradigms were created using PsychoPy (*60*) and presented on a light-emitting diode (LED) screen (Dell P2417H, 1920 by 1080 pixels) with dimensions of 54 cm by 31.5 cm. Visual stimulations consisted of a white equilateral triangle on a black background presented in different positions centered on a three-by-three rectangular grid (fig. S5) for 2 s with a 5-s black-screen interstimulus interval. The color of the stimuli and background was chosen to maximize contrast. The size of the stimulus (9 cm side) and the distance between the standard and oddball stimuli (grid distance of Δ*x* = 15 cm and Δ*y* = 9 cm) were chosen such that the stimulus was large enough to be salient but small enough to be clearly separable from other locations on the rectangular grid. The abstract triangular shape was chosen for simplicity in accordance with previous studies in vision research (*61*). In a smaller set of experiments, we used a more naturalistic stimulus on the screen to verify our results under these circumstances. A picture of the home pond of the animals was used as a permanent background, and the head of a conspecific turtle appeared on various positions on the pond (same positions on the screen as in the previous experiment). We used an oddball paradigm. The “standard” stimulus position in these experiments was presented 10 times more frequently than the “deviant” position. The positions were chosen from five different positions (the four corners and the middle of the screen) and were varied between and within single animals (fig. S4). To ensure synchronization between the visual stimuli and the electrophysiological data, digital triggers were sent from the computer through the PsychoPy software to the Open Ephys acquisition system. In addition, a photodiode (VTB8440BH, Excelitas) was fixed to the screen corner to report the change in screen brightness on stimulus appearance. This information was fed into the Open Ephys acquisition system via an analog signal. Combining these two methods ensured precise synchronization of the stimulus presentation. The properties and identity of each stimulus trial were recorded and saved as a CSV (comma-separated values) file by PsychoPy.

For each oddball experiment, a spatial control session was performed in which stimuli in different locations were shown with equal probabilities. Specifically, a rectangle (7.5 cm by 7.5 cm) was shown randomly in either one of five (center and four corners) or nine different positions on a three-by-three rectangular grid (fig. S5C) with equal probability. The positions were the same used in the oddball experiment. A rectangular stimulus shape was chosen to prevent possible association with the triangles in the oddball experiments. Each position was presented 20 times for 2 s with a black-screen interstimulus interval of 5 s. In the naturalistic oddball experiments (turtle stimuli), the same parameters as in the abstract stimuli were used (same timing and the same nine positions), but the stimuli consisted of a turtle head appearing on a background of a pond.

For each animal, multiple control and oddball experiments were performed sequentially over several days with the same or a different combination of standard and deviant position. Due to the response adaptation observed between sequential experiments, we analyzed only the session in which a new combination of positions was shown for the first time resulting in 50 sessions from 11 animals. The naturalistic oddball experiments were performed on a different set of animals. The last eight sessions for each animal were included in the analysis before the quality of the recorded signals decreased and after the animal recovered from the surgery and achieved steady-state behavior, resulting in 24 sessions from three animals.

### Statistical analysis

All statistical analyses were performed in MATLAB (R2024b, MathWorks) using custom-written scripts, if not mentioned otherwise. Sample sizes were selected according to common practices in the field, taking into account the nature of the experiments performed and the availability of the nonconventional animal species studied. Results were replicated across independent experiments with the exact number of statistical replicates (within and between animals) noted in the figure legends and text. In all box plots, the square represents the interquartile range (IQR), the middle bin is the median, and the “whiskers” extend to points that lie within 1.5 IQRs of the lower and upper quartile. Correlations between head and eye angles were calculated using Pearson correlation and visualized by a least-squares regression line. Permutation tests and significance calculations are described below.

### Response intensity (PC1) calculation

In most recordings, the LFP response amplitudes of standard and deviant were different. However, since response patterns (amplitude, width, timing, etc.) varied considerably across animals (possibly due to variations in the exact cortical recording location), the response differences between the standard and deviant stimuli were quantified by the amplitude of PC1 following principal components analysis (PCA). This approach has the advantage of evaluating the magnitude of response variability across animals using the same parameter. To calculate PC1, time windows around the response to the visual stimuli (from 80 to 580 ms post–stimulus onset) were extracted from the raw data (fig. S2A). A small minority of trials with artifacts (∣voltage∣ > 900 μV) were removed from the analysis. The data were downsampled (400 Hz) resulting in 200 data points for each response curve that were fed into the PCA.

To avoid biasing results toward the more abundant standard trials, the PCs were calculated for an equal number of standard (20 randomly selected) and deviant trials (all 20 trials). Due to the high response variability and low number of deviant trials, we increased robustness by repeating this procedure 100 times with different randomly selected sets of 20 standard trials and calculating the average PCs. Last, the entire population of trials (200) was projected onto these PCs (fig. S2B). For simplicity, only the first PC was used for all further analyses. We note that deviant and standard could be separated (although to a lesser extent) also using higher PCs (Fig. 1I).

In the control experiments in which positions were shown with uniform distribution over locations, we projected the data on the PC1 of the corresponding oddball experiment. The direction of the response on the PC axis plays a role in this case, as a response to a specific position on the screen might occur in the opposite direction of the novelty response (fig. S5E). To account for this effect, we flipped the PC scores of all sessions where the novelty effect was negative and tested the responses to the different positions in the control as well as the oddball session. If subtracting the response to the control stimulus from the oddball stimulus led to it falling below a significance level (*P* > 0.05), these sessions were marked in red (fig. S5F).

### Permutation tests

To test for significant differences between responses to standard and deviant stimuli (fig. S2A) in each recording session, we used a permutation test (*62*). First, we extracted the PC1 scores from all standard and deviant trials (fig. S2B). Next, we randomly shuffled (10,000 permutations) trial identities and calculated the distances between the means of 20 random “deviant” trials and the standard trials (excluding the first 10 trials; fig. S2C). The distribution of these distances (fig. S2C, blue bars) was compared to the measured distance in the experiment (fig. S2C, red dashed line) to evaluate the probability of measuring such distance by chance. When performing the same procedure for calculating the significance of the difference between the first 10 trials in a session and standard trials, only 10 random trials were used in the permutation test.

To calculate the significance of all sessions across animals, the distance between the mean of the standard and the mean of the deviant responses was normalized to the standard deviation of the standard stimuli PC1 distribution following the permutation test. Specifically, the normalized (*z*-scored) PC1 was calculated as nPC1 = (*m*PC1*_X_* − *m*PC1_Standard_)/*s*PC1_Standard_, where *m* and *s* are the mean and standard deviation, respectively, and *X* is the trial group (e.g., Fig. 1K: Standard; Deviant; First10 stimuli; and 1After, the first stimuli following the presentation of a deviant). nPC1 values greater than 2 indicate that the differences in the specific session were significant (*P* < 0.05). We used the same permutation test (*62*) to calculate the significance of comparisons between groups across animals and sessions (Figs. 1, K and N; 2, E, F, and I; and 3C) by shuffling the identity of sessions in the two compared groups (10,000 permutations).

### Spike sorting

In a small subset of recordings, we were able to record spiking activity in addition to LFP. In these recordings, spikes were detected, extracted, and sorted using Kilosort 3 and manually verified using Phy (*63*). Raster plots were generated using the “plotSpikeRaster” (*64*). We did not perform any statistical analysis on these data.

### Top camera

A top camera (FLIR Flea3, Teledyne) above the animal recorded the animal’s movements at 15 Hz throughout all experiments using SpinView software (v. 2.7.0.128, FLIR). Its acquisition was controlled by an Arduino board (Arduino Nano Every) via a custom-made script. In addition to sending triggers to the camera to acquire frames, triggers were sent to the acquisition board to synchronize the camera frames with the electrophysiological recording. To ensure the precise synchronization of the video frames with the electrophysiological recording, videos were manually examined to cross-validate the time of stimulus appearance in the video with its appearance in the photodiode signal (measuring frame changes on the visual stimulation screen) recorded by the data acquisition system.

### Head angle extraction

To extract the head positions from the top camera videos, two points on the rostral-caudal sections of the head were labeled and tracked using DeepLabCut software (v. 2.3.5) (*65*). The head position for each trial was calculated as an average position of the pixel at the center of the head during the frames between the visual stimuli onset and 400 ms poststimuli. A zero angle was defined as the average angle across trials, and a positive head angle indicated head turns to the right. To test the link between the head angle and the neural responses, the trials were divided into left (Θ < −15°) and right (Θ > 15°) head angles, relative to the mean of all angles. This ensured that “left” and “right” viewing represented groups of different angles. To identify trials in which the stimulus was seen from a certain angle for the first time, a distance matrix was applied to the head angles at each stimulus presentation. Trials that had a distance of at least 5° from all previous trials were marked as the “first time viewing.” For this analysis, we focused on the sessions in which response intensities to deviant stimuli were significant (*P* < 0.05; Fig. 1K). In addition, we excluded recordings in which turtles viewed stimuli without extending their heads out of the carapace, making tracking of the head impossible, and one recording in which the video file was corrupted, leaving 23 sessions from eight animals for analysis.

### Eye camera system

To track the eye movements and estimate the gaze vector, we designed an ultrasmall camera system adapted from Meyer *et al.* (*66*). The system was mounted on a thin aluminum beam screwed into a groove in the dental cement. This system was mounted before the start of the recording sessions and allowed the animals to fully retract their heads into the carapace during experiments. The weight of the system was balanced using a pulley. The system consisted of an adjustable 3D-printed camera holder that was connected to the aluminum beam. The camera holders consisted of two joints, allowing precise calibration of camera angles (Fig. 2F). To minimally obstruct the visual field of the animal, the cameras (5MP OV5647 Miniature Camera Module, Arducam) were pointed toward infrared (IR) mirrors (transparent at visible wavelengths) extending from the camera mount (Fig. 2F). IR LEDs (VSMY2943G, Vishay) were mounted on the holders to provide constant illumination. Each camera was driven to acquire images by a Raspberry Pi (3B+) via custom Python scripts. In addition to acquiring the videos, digital triggers for each frame were sent from the Raspberry Pi digital port to the Open Ephys acquisition board. Videos were recorded simultaneously from both eyes at 60 Hz. To verify and, if necessary, adjust the synchronization of the eye videos to the Open Ephys acquisition board, the IR LEDs that were used to illuminate the eyes were turned off once every minute by their LED driver [Cyclops LED driver, Open Ephys; (*67*)], resulting in one dark frame in the videos of the eyes. Simultaneously, digital triggers for each LED blink were sent from the LED driver to the Open Ephys acquisition board, which was used to verify the timing of the dark frames.

The pupil outline was labeled and tracked using DeepLabCut software (v. 2.3.5) (*65*). An ellipse was fitted (*68*) to the pupil outline, and its midpoint was used as the position of the pupil (*x*, *y*). The edge of the eye socket was labeled manually, once for each recording session, and its radius and center were calculated by fitting a circle on the outline data points. To calculate the eye angles relative to the center of the eye socket, all points were shifted to make the midpoint of the eye socket the origin (0, 0). The angles that the pupil was pointing at in *x* and *y* directions were estimated using the arccosine: $\text{angl}e_{x}=\text{arccos}\left(\frac{x}{\text{radiu}s_{\text{socket}}}\right)$. As the center of the eye socket did not align with the regular position of the pupil, the eye angles (ψ) were calculated relative to the mean over all eye positions. To account for the head tilt, the horizontal axis was set as the axis through the visual streak of the eye (visible in Fig. 2G). This line is known to be aligned with the horizon most of the time (*69*). The angle φ in this new horizontal direction was calculated by using a rotation matrix (*R*) on ψ in *x* and *y* directions, where ρ is the angle of visual streak relative to the *x* axis of the video, R=[cos(ρ),−sin(ρ);sin(ρ),cos(ρ)], and $\left[\varphi_{x},\varphi_{y}\right]=R\times \left[\psi_{x},\psi_{y}\right]$. φ was estimated based on the assumption that the eye is a round sphere centered within the eye socket. The reported range of eye angles in this study lies within the range of values reported previously from head-fixed turtles (*70*) and freely moving birds (*71*). To calculate the viewing angle, the corresponding eye and head angles were added linearly. To test whether spatial selectivity was dependent on a combination of viewing angle and head angle, we recorded and analyzed 12 oddball sessions with significant responses to the oddball from three animals.

### Head and eye movements following visual stimulation

The turtle’s movement in response to the deviant stimulus could possibly lead to DC activation (e.g., due to a startle response). To test this, we examined DC response intensity (PC1 as in Fig. 1I) as a function of average head and eye speeds following visual stimulation (calculated during 80 to 480 ms post–stimulus onset) (fig. S9). Head speed was calculated from the nose position (see head angle extraction). Eye speed was calculated from the pupil center. Paired *t* tests were performed between the mean speed of standard and deviant trials within each recording session. The same sessions as in Fig. 2 were used for this analysis (*N* = 23 from eight animals for head movements, *N* = 12 from three animals for eye movements).

### Comparison of responses between head or eye movement and visual stimulation

Movement onset was detected by using the “findpeaks” function (MATLAB) on the head and eye movement speeds. This allowed us to detect isolated peaks and excluding long episodes of movement without clear peaks. To compare responses during head and eye movement with responses during visual stimulation, we calculated the activity magnitude: the average of the absolute LFP during the response window. We used a 200-ms window, targeting the initial response to visual stimuli. For visual stimuli, this window started 80 ms after stimulus onset. Because movement epochs were not time locked to a temporally accurate event (visual responses could be elicited during the entire movement epoch), we used a sliding window of 200 ms (50-ms steps), starting at movement onset and ending 1 s after, to find the window with the largest response. As eye movements were generally shorter than head movements, we limited the sliding window to 0.5 s after movement onset capturing the full movement duration. Because movements appeared throughout the presentation of visual stimuli, we excluded episodes after the onset and the offset of visual stimuli (80 to 480 ms).

In addition, the sliding window analysis was performed on longer windows, thus increasing the probability of capturing spontaneous cortical excitations unrelated to movement. To account for this effect, we normalized the results by subtracting the baseline activity magnitude by applying the same approach to calculate the activity in the closest episode that did not contain movement (or visual stimulations). This was done for each epoch separately. These results were compared to the normalized (baseline-subtracted) visual stimulations. In this case, the baseline was calculated on a 200-ms window prior to the visual stimulus onset. To calculate statistics over sessions, we subtracted the mean response size to head or eye movements from the mean response size to visual standard and deviant stimuli in each session (Fig. 3D). A *t* test was performed on the population to calculate the probability of them being greater than zero. All recording sessions with significant responses to the deviant (*N* = 28; Fig. 1K) were included in this analysis, except for one recording with a corrupted video file leading to 27 recording sessions from nine animals.
