# Supplementary material for: View-invariant representations in ancestral cortex
Source: Sci Adv. 2025 Nov 26;11(48):eady9659. doi: 10.1126/sciadv.ady9659 (PMC12652236; doi:10.1126/sciadv.ady9659)
Supplement: Supplementary file 1 — Figs. S1 to S10 Legend for movie S1 [file sciadv.ady9659_sm.pdf]

Science Advances
AAAS

Supplementary Materials for

View-invariant representations in ancestral cortex

Milan Becker *et al.*

Corresponding author: Mark Shein-Idelson, sheinmark@tauex.tau.ac.il

Sci. Adv. **11**, eady9659 (2025)
DOI: 10.1126/sciadv.ady9659

The PDF file includes:

Figs. S1 to S10
Legend for movie S1

Other Supplementary Material for this manuscript includes the following:

Movie S1

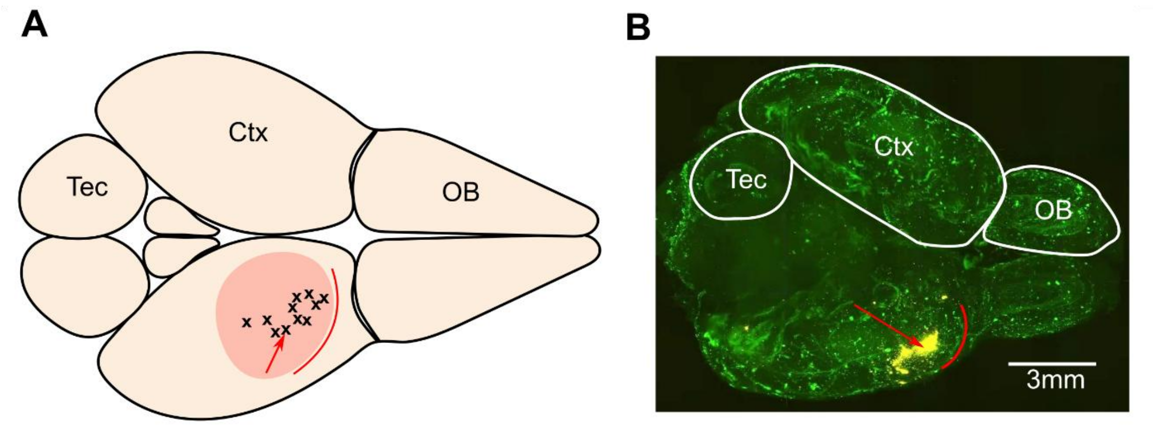

Fig. S1. **Verification of recording locations.** (**A**) A schematic drawing of a top view of the turtle brain with insertion sites marked (x) across animals. Red shaded area marks DC. (**B**) A top view (maximal projection image) of a cleared turtle brain (green, tissue autofluorescence) acquired using a light sheet microscope. The electrode insertion site was stained by the cm-DiI (yellow) coating the electrode. Red arrow marks the insertion site, red line marks the rhinal fissure used for orientation throughout insertions. Tec=Tectum, Ctx=Cortex, OB=Olfactory bulb.

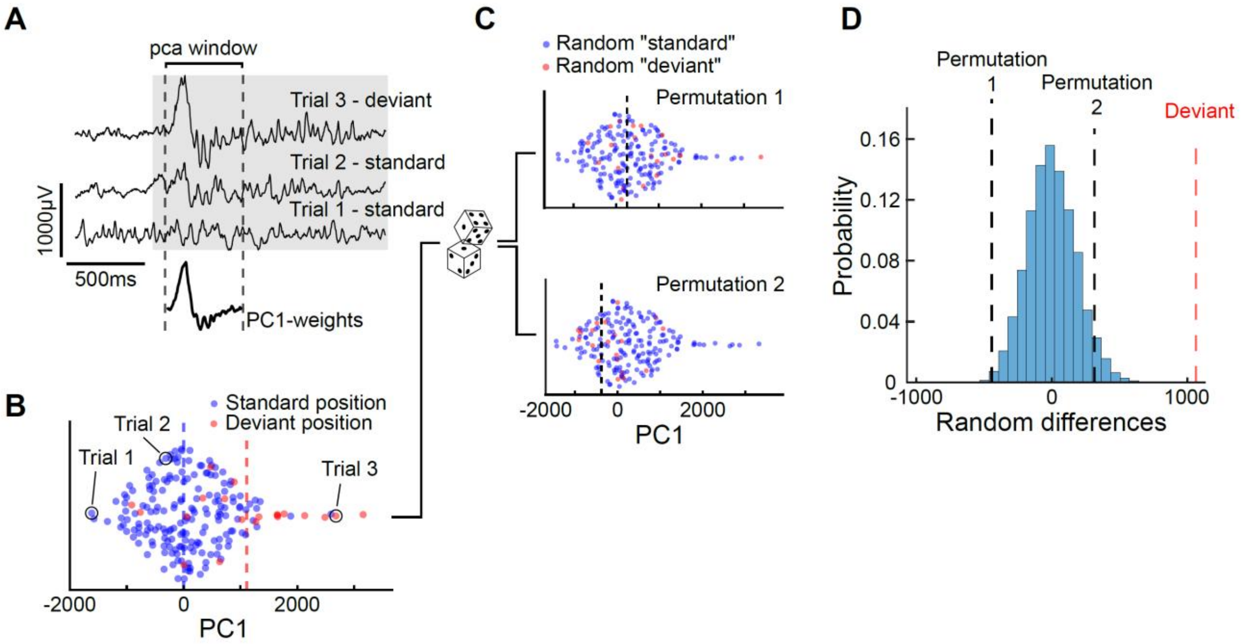

Fig. S2. Permutation test used to quantify differences between standard and deviant stimuli LFP responses within each session. (**A**) LFP of three example trials with high (trial 3, response to a deviant stimulus), intermediate and low (trials 1,2, responses to standard stimuli) response magnitudes. Shaded area marks the visual stimulus, and dotted lines show the time window used for PCA analysis. Bottom: the first PC (PC1) weights for this session. Note that the weights correspond to the LFP response curves (top). (**B**) The PC1 values (randomly distributed on the vertical axis for visibility) for the same recording session (trials shown in (A) are marked). Note that stronger responses correspond with higher PC1 values. The means for standard (blue) and deviant (red) trials are marked with dashed lines. (**C**) Two examples of the PC1 values (as in (B)) after shuffling the stimulus identity (permutations 1 and 2). The means of the randomly selected "deviant" trials are depicted by dashed lines. (**D**) The distribution of means for the deviant trials for all permutations (N=10000). The two permutated averages in (C) are marked by black dashed lines. Note that the value for the real deviant trials (red dashed line) lies well outside the values of permuted trials.

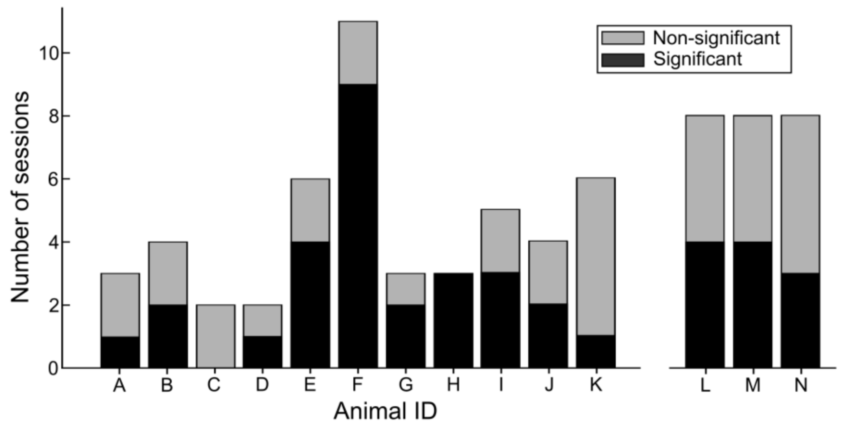

Fig. S3. **Most animals exhibit significant spatial selectivity.** The distribution of the number of sessions with significantly different responses (black, $p<0.05$; permutation test calculated as in Fig. 1) between the standard and deviant stimuli across animals (28 out of 50). Notice that most animals (10/11) showed at least one significant session. Animals L-N were used solely for the naturalistic stimulus experiments (3 animals, significant sessions: 11 out of 24).

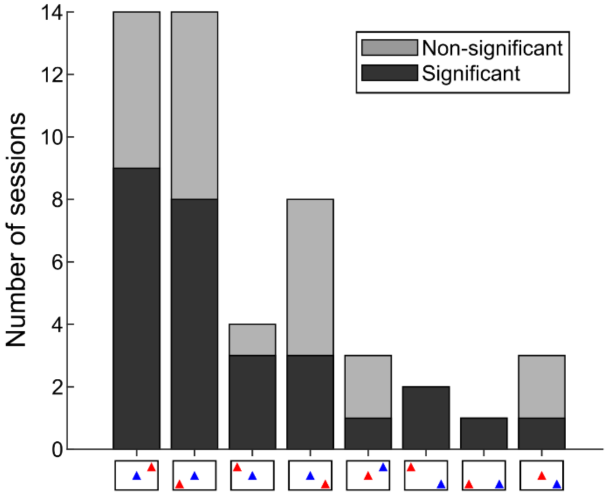

Fig. S4. **Spatial selectivity is observed across different standard and deviant locations.** Distribution of the number of sessions with significantly different responses (black, $p<0.05$; permutation test calculated as in Fig. 1) between the standard and deviant stimuli across different standard (blue) and deviant (red) positions on the screen. Notice that all tested positions exhibited spatial selectivity.

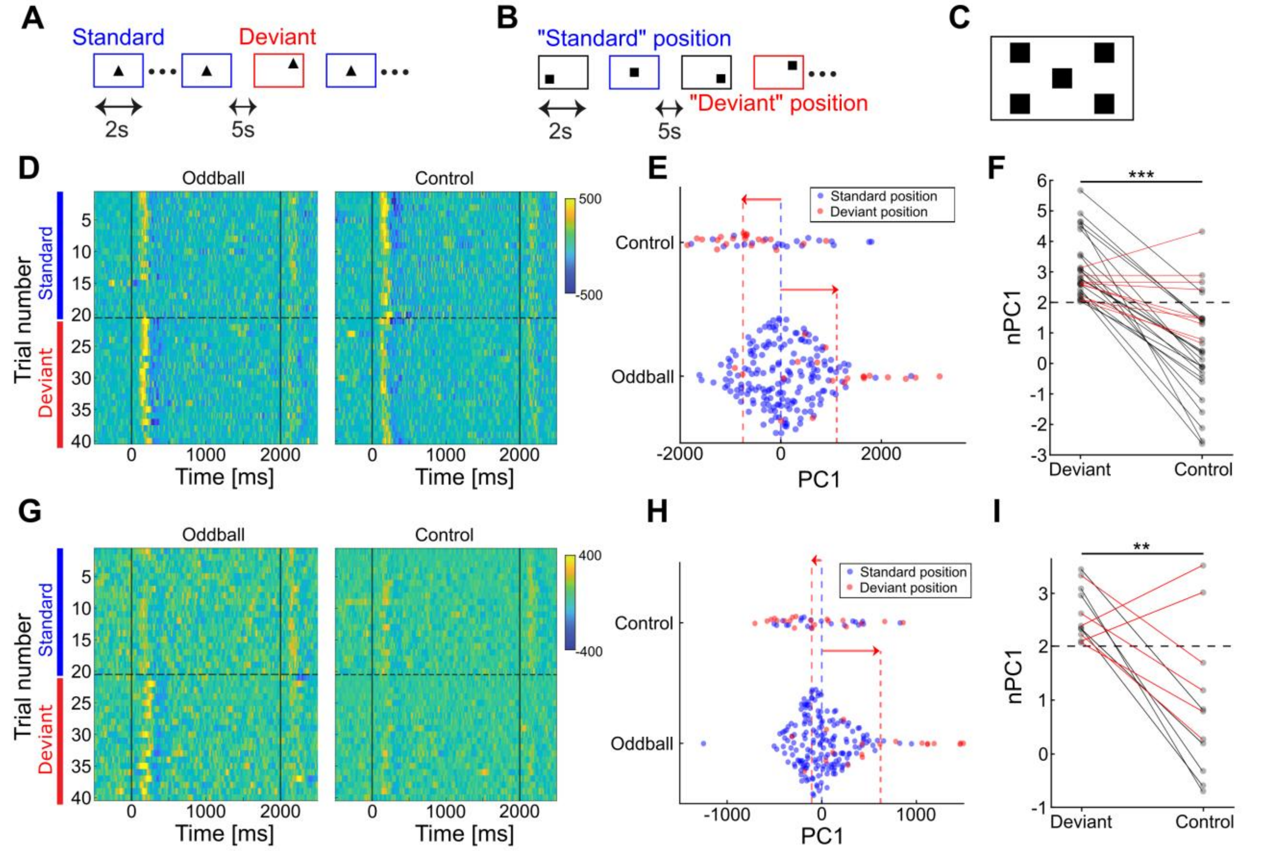

Fig. S5. **View-invariant adaptation is not explained by biases to stimulus positions.** (**A**) Schematic representation of the oddball experiments in which the deviant position is shown in a ratio of 1:10. (**B**) Schematic representation of a control experiment in which all positions are shown randomly with the same probability (20 stimulations per position). To compare these experiments with the ones in (A), the "Standard" and "Deviant" positions are defined according to the oddball experiment (A) conducted afterwards. (**C**) All stimulated positions on the screen. (**D**) Comparison between LFP responses (as in Fig. 1G) for stimuli in the same location during an oddball session (left, shown are 20 standard trials and the 20 deviant trials) and a uniform position probability control session (right). The top and bottom traces (divided by dashed line) are responses to the standard, and deviant stimulus position, respectively. Notice that while differences in responses to different locations may exist, these differences are not the same as the differences observed following adaptation during the oddball experiment. (**E**) The PC1 score (as in Fig. 1I) for the oddball (bottom) and control experiment (top, as in (D)) during one recording session (the same standard, blue, and deviant, red, positions were stimulated). The average of the deviant (right dotted line) is significantly ($p<0.01$, permutation test) different from the normal position. In contrast, the "deviant" average in the control experiments have negative values. (**F**) Normalized differences (as in Fig. 1K) between the deviant position in the oddball and control experiments. This comparison was performed for all significant novelty experiments and the corresponding control experiments with random positions. For clarity, the direction of the deviant response in the oddball session was defined as positive. Red lines indicate oddball experiments that could be

explained by a position bias (9/28 sessions). (**G-I**) Same as (D-E) but for the naturalistic stimulus experiments. Note that the same stimulus shape (turtle head) was used in both oddball and control experiments. Pooling over animals (I) indicates that the differences between oddball and standard stimuli are significantly higher than in control experiments (permutation test; $p<0.002$, $N=11$ from 3 animals). Therefore, even though some oddball responses fall below the significance level following the subtraction of the control stimulus (6/11 sessions, red lines), the novelty response cannot be explained by a bias to position.

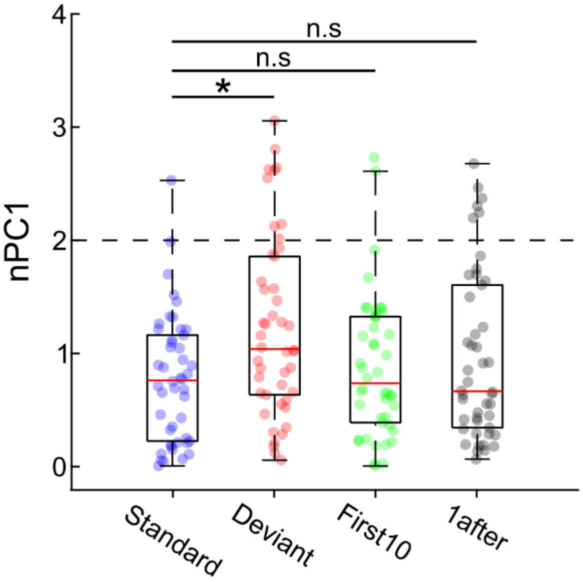

Fig. S6. **Off responses show limited spatial selectivity.** Summary statistics over all recording sessions (N=50 from 11 animals) as in Fig. 1K, but for the responses to the offset of the stimuli. Shown are the normalized average PC1 values for the following trial groups: deviant trials, 20 randomly selected standard trials, first 10 trials and the first trials after the deviant presentation. Each data point is z-scored as follows: $nPC1=(mPC1_X-mPC1_{Standard})/sPC1_{Standard}$, where m and s are the mean and standard deviation, respectively, and X is the trial group. In contrast to the responses to stimulus onset, there was only a small, but significant (permutation test, $p<0.01$), difference between the responses to the standard and the deviant. There were no significant differences between the standard stimuli and the first ten stimuli or the 1st stimuli after the deviant (permutation test, both $p>0.1$).

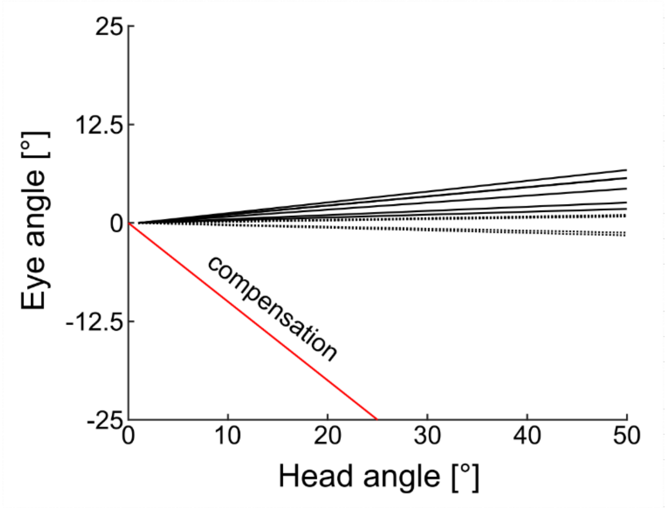

Fig. S7. **Head angles are not compensated for by eye angles.** Linear fit slopes (least-squares) for all recorded sessions. In each session the head and eye angle values for each trial were used for calculating the fit (as shown in Fig. 2H). In addition, Pearson correlation was calculated for each session with significant ($p<0.05$) correlation values in solid lines. Though there were weak correlations in some sessions, these correlations were small and positive. Further, linear fits revealed small slopes that did not indicate a compensation of head and eye angles (N=12 from 3 animals). Red line represents a hypothetical slope indicating full compensation.

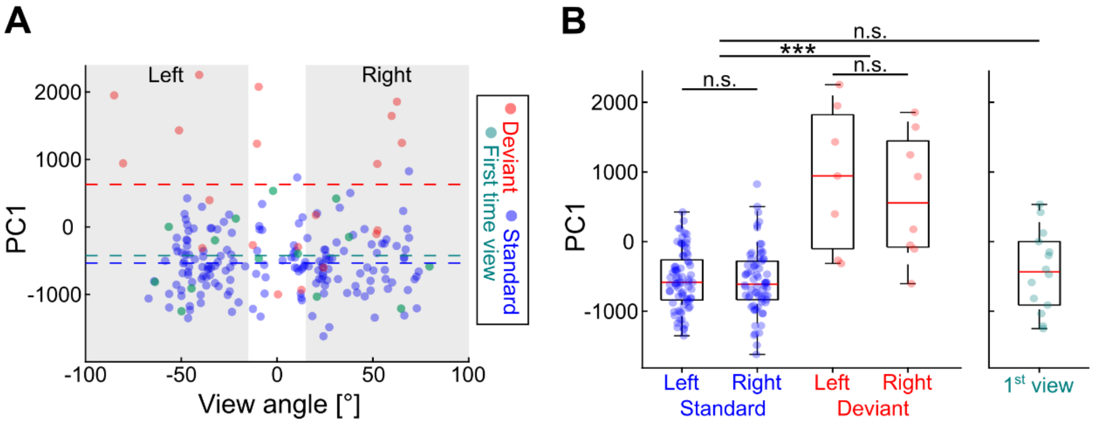

Fig. S8. **Spatial selectivity is independent of the combined head and eye viewing angle.** (**A**) Response intensity (PC1, as in Fig. 1I) as a function of combined head and eye viewing angle. Blue - standard stimuli, red - deviant stimuli, green - trials in which a new angle was viewed the first time (1st view). 0° is defined as the average angle. Notice PC1 amplitude significantly differs between standard and deviant but not between different head angles. Shaded grey marks grouping to left viewing angles ($\Theta < -15°$) and right viewing angles ($\Theta > 15°$). (**B**) PC1 for standard, deviant, 1st view, $\Theta < -15°$ (left), and $\Theta > 15°$ (right) group trials. Notice no significant differences between left and right in standard or deviant trials ($p > 0.9$, permutation test) or standard and 1st view trials ($p > 0.7$, permutation test), but a significant difference between standard and deviant trials ($p < 0.001$, permutation test).

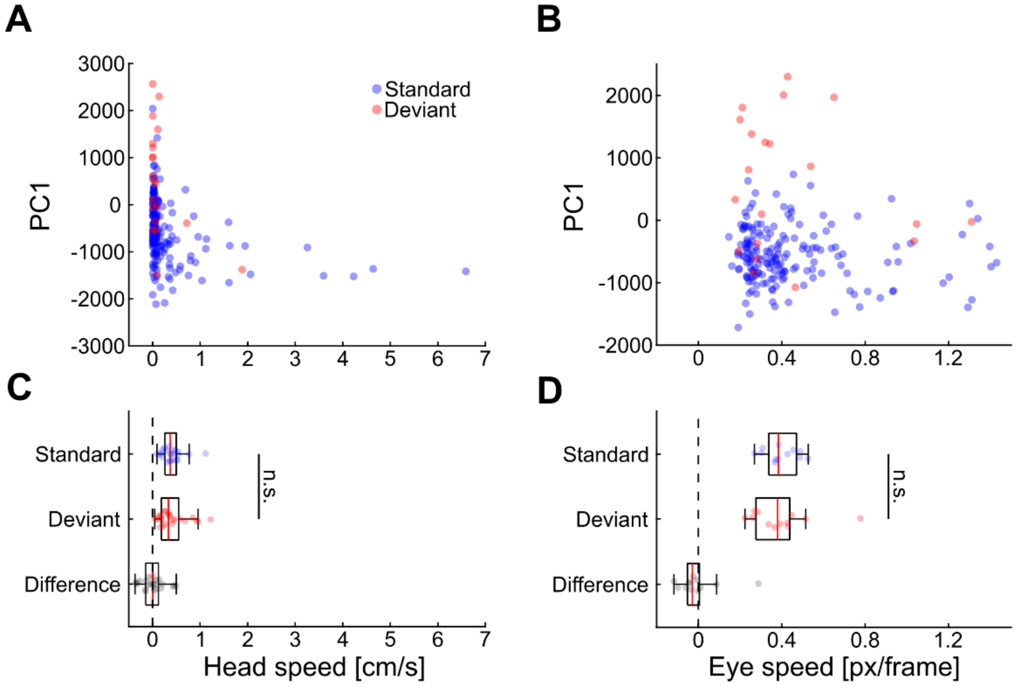

Fig. S9. **Deviant stimuli do not elicit different head or eye movements relative to standard stimuli.** (**A**) Cortical response intensity (PC1 as in Fig. 1I) as a function of head movement speed during single trials (red, deviant trials; blue, standard trials; averaged over 80-480ms post stimulus onset). Notice no increase in movement during deviant trials. (**B**) Same as (A) but for eye movement speeds. (**C**) Head speeds averaged over all standard trials (blue), deviant trials (red), and the difference between standard and deviant trials (grey) for different experiments (N=23 from 8 animals). There were no significant differences in head speeds between standard and deviant stimuli (p>0.8, paired t-test). (**D**) Same as (C), but for eye movement speeds (N=12 recordings from 3 animals). There were no significant differences in eye speeds between standard and deviant stimuli (p>0.9, paired t-test).

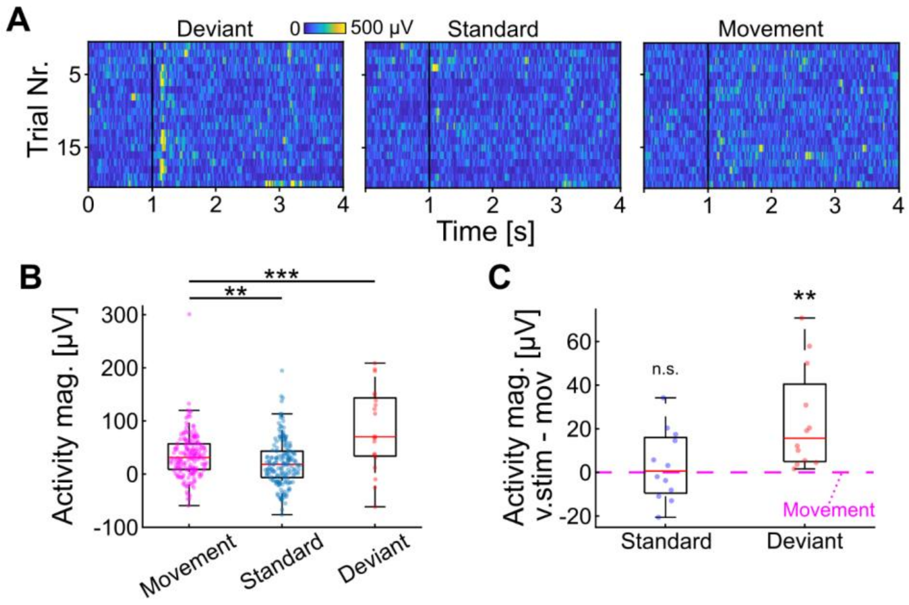

Fig. S10. Eye movements elicit weaker cortical responses than visual stimulation. (**A**) Heatmaps showing absolute LFP responses over trials, shown are 20 random trials for movement (right), standard stimulus (middle) and the 20 trials of the deviant (left). (**B**) Relative activity magnitude, calculated as mean absolute LFP amplitude above baseline for movement, standard stimuli and deviant stimuli across trials. Notice differences between movement and deviant conditions ($p<0.0002$, permutation test). The standard stimulus did generally elicit very weak responses in this example session resulting in weaker responses compared to the movement ($p<0.004$, permutation test). (**C**) Relative response magnitude of standard and deviant stimuli after subtracting the responses to movement (pink dashed line) average in each recording session. The deviant stimuli elicited consistently stronger responses relative to movement, in contrast to the standard stimulus, which was comparable to movement epochs (deviant: $p<0.005$, standard: $p>0.5$, $N=12$, t-test).

Movie S1. Top camera view during an oddball experiment with head direction and LFP measurements. Top camera view during an oddball experiment (as in Fig. 2A,C). Head direction is extracted from the labels of two points on the head using deeplabcut (*65*) (red line). The visual stimulus consists of a triangle appearing in the standard position (screen center) and once in every ten trials (on average) in the deviant positions (screen top corner). The estimated head angles (middle) and LFP trace of neural activity (bottom) are shown. The shaded areas mark the times with visual stimulation (blue – standard, red – deviant). Note the responses to standard stimuli are consistently smaller than to deviant stimuli, despite head movement throughout the experiment.
